# The Value of Point-of-care Ocular Ultrasound in Physician-in-triage Model: A Case Series

**DOI:** 10.5811/cpcem.50714

**Published:** 2026-04-07

**Authors:** Christopher Thom, Benton Spirek, Gitansh Bhargava, James Moak

**Affiliations:** * University of Virginia Health System, Department of Emergency Medicine, Charlottesville, Virginia; † University of Arkansas for Medical Sciences, Department of Emergency Medicine, Little Rock, Arkansas

**Keywords:** physician in triage, central retinal artery occlusion, ocular ultrasound, case series

## Abstract

**Introduction:**

Physician-in-triage (PIT) models have become increasingly common in emergency medicine. The goal is to facilitate rapid patient evaluation and improve key operational emergency department (ED) metrics. However, there is limited time for the PIT encounter, which often involves an abbreviated patient evaluation. Point-of-care ultrasound (POCUS) has been shown to improve patient care and speed diagnosis in a variety of scenarios. Although physicians working within a PIT model must remain mindful of time constraints, POCUS can help identify time-sensitive diagnoses and guide appropriate initial testing during certain encounters. Ocular POCUS can be particularly impactful on timely diagnosis and appropriate deployment of ED resources.

**Case Series:**

We present three cases of acute monocular vision loss wherein the PIT physician used ocular POCUS to arrive at the correct initial diagnosis. This led to the appropriate deployment of ED stroke and neurology resources in the case of acute central retinal artery occlusion, while avoiding this unnecessary use of these resources in two cases where it was not indicated.

**Conclusion:**

Use of point-of-care ultrasound in PIT models should be thoughtfully employed in cases where immediate diagnosis is required, as well as when POCUS results will likely alter subsequent diagnostic testing pathways. Ocular POCUS in PIT can rapidly differentiate neurologic causes of monocular vision loss from primary ophthalmic conditions.

## INTRODUCTION

The physician-in-triage (PIT) model has seen increased deployment in emergency medicine (EM) practice. The proposed benefits include rapid identification of life-threatening or significant ailments that may benefit from immediate intervention.^1^ This model has been associated with reductions in left-without-being-seen and length-of-stay (LOS) metrics.^2^ The role of point-of-care ultrasound (POCUS) within the PIT model is not fully understood. Point-of-care ultrasound has been shown to reduce LOS in a variety of scenarios and to expedite clinical care for time-sensitive pathologies such as aortic dissection and ruptured ectopic pregnancy.^3^ However, it also takes time to perform, and the PIT evaluation must be brief to facilitate efficient evaluation of patient arrivals. Maintaining this balance remains a challenge for those emergency physicians working in a PIT role who also aim to deliver excellent clinical care.

Time-sensitive visual complaints require rapid and accurate diagnosis to facilitate favorable outcomes.^4^,^5^ Physicians in triage will often see patients in vertical chairs with lighting and room ergonomics that are not conducive to thorough examination techniques. One particularly time-sensitive pathology is central retinal artery occlusion (CRAO). While controversial, prompt administration of thrombolytic therapy could possibly lead to improved visual outcomes.^4^ The workup of these patients is often facilitated by a stroke alert system in the emergency department (ED), but this can expose patients to unnecessary cost and radiation when not warranted.

Research is limited on the use of POCUS to diagnose ocular conditions within the PIT model. We present three cases of acute monocular vision symptoms presenting to the ED and evaluated by the physician in triage. In each case the physician employed ocular POCUS, which led to the correct initial diagnosis and management plan. These cases highlight the importance of thoughtful employment of ocular POCUS within the PIT model.

## CASE SERIES

### Case 1

**Image 1:**
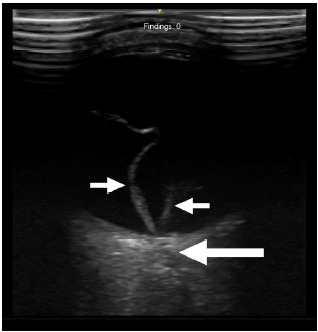
Point-of-care ultrasound demonstrating retinal detachment. The small arrows denote the retina having detached from the posterior globe. The large arrow denotes the optic nerve sheath.

A 33-year-old male with a history of sickle cell disease presented with the acute onset of left monocular vision changes. He described a sudden onset of painless flashes, followed by a “darkening” of the vision in his left eye one hour prior. On PIT evaluation, the pupillary response to light was intact, and his conjunctiva appeared normal. Visual acuity was finger counting only in the left eye and 20/30 in the right eye. The PIT physician performed ocular ultrasound and identified a retinal detachment (Image 1). Given this finding, a stroke alert was not activated, and the patient was sent to a dedicated room for ocular examination within the ED. Ophthalmology was consulted and confirmed the diagnosis of retinal detachment (macula-off).

*CPC-EM Capsule*

What do we already know about this clinical entity?

*Ocular point-of-care ultrasound (POCUS) allows for rapid diagnosis of patients presenting with acute monocular vision change, but its use has not been fully explored*.

What makes this presentation of disease reportable?

*This case series highlights the use of ocular POCUS at triage to rapidly differentiate neurologic from ophthalmic etiologies*.

What is the major learning point?

*The immediate diagnosis provided by ocular POCUS at triage can be consequential, particularly in the case of central retinal artery occlusion*.

How might this improve emergency medicine practice?

*Thoughtful use of POCUS at triage is essential for timely diagnosis of critical, time-sensitive conditions and should be encouraged in such scenarios*.

### Case 2

**Image 2:**
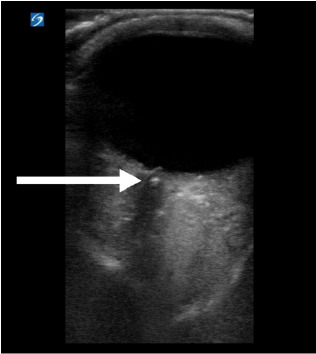
Point-of-care ultrasound demonstrating a central retinal artery occlusion. The arrow indicates the retrobulbar spot sign within the optic nerve sheath.

A 72-year-old male with a history of hypercholesterolemia and cataract surgery presented with acute onset of left monocular vision changes. He reported floaters in his left eye 90 minutes prior to arrival with accompanying blurry vision. He denied associated headache, fever, vomiting, or eye pain. On PIT evaluation, the conjunctiva appeared normal, the pupillary response to light was normal, and there were no neurological abnormalities on exam. Visual acuity was finger counting only in the left eye and 20/20 in the right eye. The physician in triage performed an ocular ultrasound and identified a retrobulbar spot sign (Image 2). Given this finding, a stroke alert was activated, and the patient was sent for an emergent computed tomography angiogram (CTA). This study was unremarkable, and the stroke neurology team then recommended thrombolytic therapy. Tenecteplase was given in the ED, and ophthalmology was consulted. A dilated eye exam by the consultant confirmed suspicion for CRAO, and he was admitted to the neurology service with a near return to vision baseline by time of hospital discharge.

### Case 3

**Image 3:**
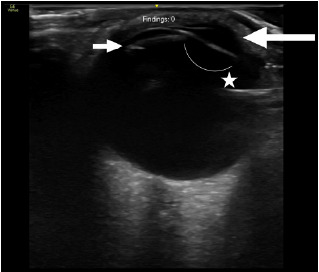
Point-of-care ultrasound demonstrating artificial lens dislocation. The large arrow denotes the anterior chamber of the eye, while the small arrow denotes the displaced lens. The correct anatomical position of the lens is denoted by the addition of the curved line adjacent to the star.

A 75-year-old male with a history of diabetes and cataract surgery presented to the ED with acute onset of right monocular vision changes. He reported onset of blurry vision in his right eye approximately three hours prior. He noted that this occurred acutely while at rest and without any accompanying symptoms. He described the vision change as a “haze” over the right eye. On PIT examination, visual acuity testing was 20/70 in the right eye and 20/20 in the left eye. Pupils were equal and responsive to light stimuli. Visual fields were grossly intact bilaterally. Ocular ultrasound was performed and revealed findings consistent with a lens dislocation (Image 3). Ophthalmology was consulted; after confirming the diagnosis, the consult recommended outpatient follow-up for corrective surgery.

## DISCUSSION

In the above cases, the physicians in triage used ocular POCUS to appropriately triage patients to the correct care pathway. All three patients presented with acute monocular vision complaints with overlapping symptoms confounding the diagnosis. With ocular POCUS, the physician was able to rapidly differentiate neurologic from ophthalmic etiologies and proceed with the appropriate downstream testing and management. The physician in triage appropriately pursued a stroke alert activation and CTA imaging for the CRAO patient, while avoiding this pathway for patients with retinal detachment and lens dislocation. Central retinal artery occlusion was strongly considered in the patient with sickle cell disease, a known risk factor for this condition.^6^ However, the identification of a definite retinal detachment on ocular POCUS correctly led the physician to triage this patient back to the available eye room in the ED to await urgent, but not emergent, ophthalmology evaluation.

While these cases could have been differentiated with more thorough examination techniques and dilated fundoscopy, this is not possible in most PIT paradigms. The PIT encounter must be brief to keep up with patient arrivals yet still enable the physician to accurately identify appropriate interventions or alerts that are needed immediately at the time of patient arrival. Point-of-care ultrasound can play a critical role in the correct identification of these patients, as there can be overlap of symptoms and exam features between CRAO and retinal detachment.^5^,^7^ As the above case series demonstrates, ocular POCUS can aid in appropriately triaging patients who might benefit from stroke alert activation.

The test characteristics of POCUS for retinal detachment have been previously well established, with studies demonstrating a sensitivity of 94% and specificity of 96%.^8^ This accuracy is high enough to be of diagnostic value for the emergency physician in a variety of contexts, particularly in ED settings without available ophthalmology consultation. In retinal detachment, the ultrasound will show a mobile, echogenic line floating within the vitreous cavity, which can be more evident when the patient moves their eye. It will appear “tethered” to the optic nerve sheath, which helps differentiate the retinal detachment from a posterior vitreous detachment.

The accuracy of POCUS in detecting CRAO has not been the subject of extensive study. In CRAO, ultrasound may show decreased or absent flow in the central retinal artery and, in a proportion of cases, the retrobulbar spot sign. This represents the presence of embolic material within the central retinal artery.^9^ The retrobulbar spot sign will appear as a highly echogenic “spot” within the optic nerve sheath just posterior to the retina. This is thought to represent the embolic clot within the ophthalmic artery, which runs within the optic nerve sheath. The finding can help differentiate sudden vision loss from temporal arteritis and CRAO, as the finding is generally absent in the former.^10^

In one small study with neurologists as the operator, the sensitivity of retrobulbar spot sign for CRAO was 83%, with a specificity of 100%.^10^ Another study found that the retrobulbar spot sign was present in 32 of 46 patients (70%) with a CRAO and that interobserver agreement among various physicians was high.^11^ Early detection of CRAO can facilitate treatment with thrombolytics, although this treatment does remain controversial. In one study, 86% of CRAO patients treated with early thrombolysis (within 4.5 hours) had significant visual improvement.^4^ Patients receiving early thrombolysis had statistically significant improvements in vision over those patients treated without thrombolysis.^4^ Additional case series have shown visual acuity improvement in 30–55% of patients receiving intravenous (IV) thrombolysis.^12^ Ultimately, more research is needed to understand which cohort of patients with CRAO might benefit from IV thrombolysis therapy.

Lens dislocation is rare, and the accuracy of POCUS in its detection has not been rigorously studied. However, the findings are likely specific when encountered, as the appearance can be easily identified on ultrasonography. In lens dislocation, the ultrasound will show a hyperechoic, oval structure that has shifted posteriorly into the vitreous chamber or anteriorly into the anterior chamber, depending on the type of dislocation.^13^ An artificial lens, as in our third case above, will feature prominent reverberation artifact. This appears as several repeating echogenic lines directly below the artificial lens.

Concerns have been raised about impacts of the cursory nature of the history and examination inherent in the PIT model. This may lead to increased reliance on additional testing and imaging as more thorough assessments are not possible. Prior work has generally not shown an increase in unnecessary imaging upon deploying PIT models,^14^ although some centers may see an association with increased imaging utilization.^15^ Thoughtful use of POCUS in the PIT model could potentially help direct more downstream diagnostic testing and imaging use. Importantly, the PIT physician has multiple critical roles with limited time availability. Point-of-care ultrasound examinations are generally brief with a median of five minutes reported previously for ocular examinations, with further efficiency among attending physicians who have ultrasound training.^16^

We would suggest that ocular POCUS can be performed even more expeditiously when targeting the patient with monocular vision loss, as the evaluation centers on the posterior chamber and potential CRAO findings of a single eye. Physician in triage-performed POCUS should be deployed when time-sensitive, critical diagnoses are under consideration. In these cases, early diagnosis can improve time to intervention and potentially lead to improved patient outcomes. For ocular POCUS, this entails targeted use in cases of acute monocular vision loss when the differential diagnosis includes CRAO.

## CONCLUSION

Given the primary role of the physician in triage in identifying emergent conditions that need immediate therapy, the use of POCUS in cases where these conditions are suspected is important. While a routine POCUS exam may not represent the best use of the physician’s time during periods of high patient volume, determining whether a stroke alert is warranted or an aortic catastrophe is present certainly does. We would encourage the thoughtful use of POCUS within PIT models, recognizing that each situation is unique. Ocular POCUS for the rapid differentiation between neurologic and primary ophthalmic causes of acute monocular vision loss is a particularly relevant tool for the physician in triage to consider.
